# Identification of prognostic relevant chromosomal abnormalities in chronic lymphocytic leukemia using microarray-based genomic profiling

**DOI:** 10.1186/1755-8166-7-3

**Published:** 2014-01-09

**Authors:** Marian JPL Stevens-Kroef, Eva van den Berg, Daniel Olde Weghuis, Ad Geurts van Kessel, Rolph Pfundt, Matty Linssen-Wiersma, Marloes Benjamins, Trijnie Dijkhuizen, Patricia JTA Groenen, Annet Simons

**Affiliations:** 1 Department of Human Genetics, Radboud university medical center, P.O. Box 9101, Nijmegen 6500 HB, The Netherlands; 2 Department of Genetics, University Medical Center Groningen, P.O. Box 30001, Groningen 9700 RB, The Netherlands; 3 Department of Pathology, Radboud university medical center, P.O. Box 9101, Nijmegen 6500 HB, The Netherlands

**Keywords:** Chronic lymphocytic leukemia, Microarray-based genomic profiling, FISH, MLPA

## Abstract

**Background:**

Characteristic genomic abnormalities in patients with B cell chronic lymphocytic leukemia (CLL) have been shown to provide important prognostic information. Fluorescence *in situ* hybridization (FISH) and multiplex ligation-dependent probe amplification (MLPA), currently used in clinical diagnostics of CLL, are targeted tests aimed at specific genomic loci. Microarray-based genomic profiling is a new high-resolution tool that enables genome-wide analyses. The aim of this study was to compare two recently launched genomic microarray platforms, i.e., the CytoScan HD Array (Affymetrix) and the HumanOmniExpress Array (Illumina), with FISH and MLPA to ascertain whether these latter tests can be replaced by either one of the microarray platforms in a clinical diagnostic setting.

**Result:**

Microarray-based genomic profiling and FISH were performed in all 28 CLL patients. For an unbiased comparison of the performance of both microarray platforms 9 patients were evaluated on both platforms, resulting in the identification of exactly identical genomic aberrations. To evaluate the detection limit of the microarray platforms we included 7 patients in which the genomic abnormalities were present in a relatively low percentage of the cells (range 5-28%) as previously determined by FISH. We found that both microarray platforms allowed the detection of copy number abnormalities present in as few as 16% of the cells. In addition, we found that microarray-based genomic profiling allowed the identification of genomic abnormalities that could not be detected by FISH and/or MLPA, including a focal *TP53* loss and copy neutral losses of heterozygosity of chromosome 17p.

**Conclusion:**

From our results we conclude that although the microarray platforms exhibit a somewhat lower limit of detection compared to FISH, they still allow the detection of copy number abnormalities present in as few as 16% of the cells. By applying similar interpretation criteria, the results obtained from both platforms were comparable. In addition, we conclude that both microarray platforms allow the identification of additional potential prognostic relevant abnormalities such as focal *TP53* deletions and copy neutral losses of heterozygosity of chromosome 17p, which would have remained undetected by FISH or MLPA. The prognostic relevance of these novel genomic alterations requires further evaluation in prospective clinical trials.

## Background

B-cell chronic lymphocytic leukemia (CLL) exhibits a highly heterogeneous clinical course, with overall survival rates varying from several months to decades. Whereas several prognostic markers, such as expression of the CD38 and ZAP70 proteins are well-established now, these markers do not allow the identification of all patients with a high risk profile. Mutation status of the *IGHV* genes and specific genomic abnormalities, such as deletion of 11q22, trisomy of chromosome 12 and loss of the 13q14 region, provide additional prognostic information [1,2]. In addition, deletion of 17p and/or the presence of a *TP53* mutation, which are both associated with a poor prognosis, identify CLL patients with the highest risk profile [2]. Conventional cytogenetic analyses result in the identification of genetic abnormalities in a relatively low percentage of patients, due to the low *in vitro* proliferative potential of CLL cells. Even after recent improvements of this technique, using CpG-oligonucleotide DSP30 and interleukin-2 (IL-2) [3], it still does not allow the detection of submicroscopic losses such as those of the 11q, 13q and 17p regions. The most common molecular-cytogenetic techniques currently used to detect these abnormalities in CLL are fluorescence *in situ* hybridization (FISH) [1] and multiplex ligation-dependent probe amplification (MLPA) [4]. However, since FISH analysis of multiple loci is relatively laborious, and since both FISH and MLPA are targeted tests providing limited views on the genomic landscapes of CLL cells, we aimed to evaluate the diagnostic efficacy of microarray-based whole genome profiling. Next to its genome-wide character, microarray-based genomic profiling also allows the detection of small cryptic copy number alterations (CNAs) and copy neutral losses of heterozyogosity (CNLOH) that remain undetected by FISH and MLPA [5,6].

The specific aim of the present study was to compare the CytoScan HD Array platform from Affymetrix and the HumanOmniExpress 12v1 Array platform from Illumina on one hand with FISH and MLPA on the other hand by employing the currently used probe panels, targeting the chromosome regions 11q22, 13q14 and 17p13 and chromosome 12 for the detection of clinically relevant chromosomal abnormalities in CLL. To ascertain which approach would be most suitable in a routine cytogenetic diagnostic setting we also evaluated their limit of detection for the identification of small CLL (sub)clones and their resolution with respect to the detection of small focal genomic abnormalities.

## Results

### Microarray-based identification of genomic abnormalities

Microarray-based genomic profiling (12 patients with the CytoScan HD Array platform only; 7 patients with HumanOmniExpress Array platform; 9 patients on both platforms) was performed on peripheral blood or bone marrow samples from 28 CLL patients (Tables 1 and 2). In 24 of these patients genomic abnormalities (CNAs and CNLOH) were identified by microarray-based profiling: 5 cases (cases 2, 8, 14, 26 and 27) showed only one aberration (all involving loss of the 13q14 region or gain of chromosome 12), 5 cases (cases 5, 6, 16, 17 and 24) showed 2 aberrations (9 CNAs and 1 CNLOH), 5 cases (cases 7, 10, 15, 20 and 23) showed 3 aberrations (13 CNAs and 2 CNLOH), and 9 cases (cases 3, 4, 9, 11, 12, 18, 21, 25 and 28) showed more than 3 aberrations (46 CNAs and 3 CNLOH). The remaining 4 cases (1, 13, 19 and 22) showed normal microarray-based genomic profiles.

**Table 1:** Overview of genetic abnormalites as determined by FISH, MLPA and microarray-based genomic profiling

**Patient ID**	**FISH (% of abnormal cells)**	**MLPA**	**Microarray-based genomic profiling**	
			**Recurrent**	**Additional**
1	Not done	Normal	Normal	
2	Not done	Loss 13q14	Loss 13q14	
3	Not done	Loss 13q14*	Loss 13q14*	Loss 6p
		Loss 17p13	Loss 17p13	CNLOH 13q
4	Not done	Loss 11q22	Loss 11q22	Gain 2p
		Loss 13q14	Loss 13q14	Loss 6p
				Loss 7q
5	Not done	Gain 12	Gain 12	Loss 1q
6	Not done 13q14	Loss 13q14*	Loss 13q14*	
	Normal 11q22			
	Normal 17p13			
7	Normal 11q22			Loss 1q
	Not done 12	Gain 12	Gain 12	
	Loss 17p13 (21%)	Loss 17p13	Loss 17p13	
8	Not done	Loss 13q14	Loss 13q14	
9	Normal 11q22	Loss 17p13	Loss 17p13	Gain 2p
	Loss 17p13 (21%)			Loss 6q
				Gain 17p11
				Loss 20p
10	Gain 8 (28%)	Loss 17p13	Loss 17p13	Gain 8
	Loss 17p13 (16%)			Gain 17q
	Normal 11q22			
11	Loss 11q22 (58%)	Loss 11q22	Loss 11q22	Gain Xq
	Normal CEP 12	Gain 12q		Loss 3q
	Loss 13q14 (28%)	Loss 13q14	Loss 13q14	Loss 11p
	Normal 17p13			Gain 12q
12	Not done	Loss 17p13	Loss 17p13	Gain 3q22
				Loss 6q
				Gain 19p
13	Not done	Normal	Normal	
14	Not done	Gain 12	Gain 12	
15	Not done	Gain 12	Gain 12	Gain 18
				Gain 19
16	Loss 13q14 (75%)	Loss 13q14	Loss 13q14	
	Normal 17p13	Loss 17p13 (focal)	Loss 17p13 (focal)	
17	Normal 11q22	Not done	Gain 12	CNLOH 2q
	Gain 12 (21%)			
	Normal 13q14			
	Normal 17p13			
18	Normal 11q22	Not done	Loss 13q14	Loss 1q
	Normal 12			Loss 4p
	Loss 13q14 (75%)			Loss 15q15 (*MGA*)
	Normal 17p13			
19	Not done	Not done	Normal	
20	Normal 11q22	Not done	? loss 13q14	CNLOH 6q
	Normal 12			CNLOH 20q
	Loss 13q14 (5%)*			
	Normal 17p13			
21	Normal 11q22	Not done	Loss 13q14	Loss 2p
	Normal 12			Loss 8q
	Loss 13q14 (60%)			Loss 10q24
	Normal 17p13			CNLOH 17p
22	Normal 11q22	Not done	Normal	
	Normal 12			
	Normal 13q14			
	Normal 17p13			
23	Loss 11q22 (77%)	Not done	Loss 11q22	
	Normal 12		Loss 13q14	
	Loss 13q14 (50%)			
	Normal 17p13			
24	Normal 11q22	Not done	Gain 12	Gain 2q
	Gain 12 (21%)			
	Normal 13q14			
	Normal 17p13			
25	Loss 11q22 (56%)	Not done	Loss 11q22	Gain 2p
	Normal 12			Loss 4q
	Normal 13q14			Loss 5q
	Normal 17p13			CNLOH 7pq
				Loss 10p
				Loss 11q
				Loss 18q
				Gain 21q
26	Normal 11q22	Not done	Gain 12	
	Gain 12 (38%)			
	Normal 13q14			
	Normal 17p13			
27	Normal 11q22	Not done	Loss 13q14*	
	Normal 12			
	Loss 13q14 (86%)*			
	Normal 17p13			
28	Normal 11q22	Not done	Loss 13q14	Loss 6q
	Normal 12		Loss 17p13	Loss 15q
	Loss 13q14 (33%)			Gain 17q
	Loss 17p13 (39%)			

*Bi-allelic loss as determined by FISH (loss of both hybridization signals), MLPA (RCN below 1) or microarray (log2 ratio below 1).

CNLOH: Copy Neutral Loss Of Heterozygosity.

**Table 2:** Details of microarray-based genomic profiling

**Patient ID**	**CytoScan HD Array**	**HumanOmniExpress Array**	**Genes in CNAs <5 Mb**	**Type of 13q14 deletion**
1	(1–22)*x*2,(XY)x1	Not done		
2	13q14.2q14.3(50,584,486-51,470,499)x1	Not done	*DLEU2 DLEU1 DLEU7*	I
3	6p21.1p11.2(41,934,382-57,160,585)x1	Not done		
	13q14.2q14.3(48,722,871-51,051,951)0 ~ 1		*RB1 DLEU2 DLEU1*	II
	13q14.11qter(43,405,208-115,095,705)x2 hmz			(bi-allelic)
	17pterp11.2(526–21,90,786)x1			
4	2pterp13.3(12,771-69,686,286)x3	Not done		
	6pterp21.33(159,975-31,799,736)x1			
	7q36.1qter(149,853,609-159,119,707)x1			
	11q13.5q23.3(76,888,344-116,354,467)x1			
	13q14q14.3(40,480,469-52,003,234)x1			II
5	1q43(234,529,702-242,132,559)x1	Not done		
	(12)x3			
6	13q14.2(50,195,826-51,850,196)x1	Not done	*DLEU2 DLEU1 DLEU7*	I
	13q14.2q14.3(50,650,988-51,342,279)x0		*DLEU2 DLEU1*	(bi-allelic)
7	1q42.12q42.2(224,729,057-231,273,551)x1	1q42.12q42.2(224,726,060-231,572,255)x1		
	(12)x3	(12)x3		
	17pterp11.2(526–21,076,299)x1 ~ 2	17pterp11.2(8,547-22,208,945)x1		
8	13q14.2(50,519,996-51,503,800)x1	not done	*DLEU2 DLEU1 DLEU7*	I
9	2pterp14(12,771-67,026,521)x2 ~ 3	2pterp14(12,771-67,026,521)x2 ~ 3		
	6q14.1q21(80,048,777-111,874,976)x1	6q14.1q21(80,134,141-111,850,742)x1		
	17pterp11.2(526–18,922,732)x1 ~ 2	17pterp11.2(15,463-22,208,949)x1		
	17p11.2(19,143,976-22,261,792)x2 ~ 3	17p11.2(19,075,156-21,591,064)x2 ~ 3	*SPECC1*	
	20pterp11.1(61,569-25,598,847)x1 ~ 2	20pterp11(75,254-25,581,424)x1 ~ 2		
10	(8)x2 ~ 3	(8)x2 ~ 3		
	17pterp11.2(526–21,439,423)x1 ~ 2	17pterp11.2(8,547-22,002,556)x1		
	17p11.2qter(21,442,422-81,041,938)x2 ~ 3	17q11.2qter(25,295,032-81,051,007)x3		
11	Xq21.33qter(95,976,563-155,233,846)x2	Xq21.33qter(96,042,106-154,821,956)x2		
	3p25.3q13.12(11,420,458-106,634,792)cth	3p25.3q13.12(11,420,458-106,613,301)cth		
	11p15.4q23.3(4,388,905-114,957,588)cth	11p15.4q23.3(4,400,801-114,958,994)cth		
	12q15qter(68,548,174-133,778,166)x3	12q15qter(68,468,711-133,777,645)x3		
	13q14.2(47,596,800-50,761,018)x1 ~ 2	13q14.2(47,314,896-51,835,485)x1 ~ 2	*RB1 DLEU1 DLEU2*	II
12	3q22.1qter(133,392,418-197,851,986)x2 ~ 3	Not done		
	6q23.2qter(133,742,371-170,919,482)x1 ~ 2			
	17pterp11.1(526–22,261,792)x1 ~ 2			
	19pterp13.3(260,912-2,638,256)x2 ~ 3			
13	(1–22)x2,(XY)x1	Not done		
14	(12)x3	Not done		
15	(12)x3	Not done		
	(18)x3			
	(19)x2 ~ 3			
16	13q14.2q14.3(49,267,418-52,710,335)x1 ~ 2	13q14.2q14.3(49,253,519-52,418,598)x1	*DLEU2 DLEU1 DLEU7*	II
	17p13.1(7,285,282-7,613,708)x1 ~ 2	17p13.1(7,208,197-7,584,400)x1	*TP53*	
17	2q31.2qter(178,126,546-242,783,384)x2 hmz	2q31.1qter(178,522,104-242,082,222)x2 hmz		
	(12)x3	(12)x3		
18	1q31.1(186,115,025-186,574,022)x1	1q31.1(186,126,099-186,576,930)x1	*TPR*	I
	4p16.3(1,817,831-3,332,468)x1	4p16.3(1,824,020-3,326,393)x1	*WHSC1*	
	13q14.2q14.3(49,834,338-51,885,720)x1	13q14.2q14.3(49,826,508-51,837,299)x1	*DLEU2 DLEU1 DLEU7*	
	15q15.1(41,726,490-42,269,397)x1	15q15.1(41,726,490-42,269,397)x1	*MGA*	
19	(1–22)x2,(XY)x1	(1–22)x2,(XY)x1		
20	13q14.2q14.3(49,743,769-51,421,152)x1 ~ 2		*DLEU2 DLEU1 DLEU7*	I
	6pterp22.3(156,975-24,464,741)x2 hmz	6pterp22.1(170,044-27,221,519)x2 hmz		
	20q11.23qter(36,212,125-62,915,555)x2 hmz	20q11.22qter(32,006,475-62,155,324)x2 hmz		
21	2p22.1p16.3(39,286,190-52,873,900)x1 ~ 2	Not done		
	8q12.1q13.3(56,845,311-71,349,857)x1 ~ 2			
	10q24(102,832,471-104,451,853)x1 ~ 2		*NFKB2*	
	13q14.2q14.3(48,564,169-51,710,957)x1		*RB1 DLEU2 DLEU1*	II
	17pterp11.1(526–22,261,792)x2 hmz		*DLEU7*	
22	Not done	(1–22)x2,(XY)x1		
23	Not done	11q21.1q23.3(99,007,487-116,429,253)x1		
		13q13.3q14.11(34,736,943-41,533,052)x1		
		13q14.2q31.1(46,831,590-81,829,786)x1		II
24	Not done	2q31.1q37.3 (178,522,104-242,082,222)x3		
		(12)x3		
25	Not done	2p25.3p11.2(18,674-88,509,321)x3		
		4q12(53,897,152-54,418,635)x1	*FIP1L1*	
		4q31.3(153,069,783-154,890,126)x1	*FBXW7*	
		5q21.2q35.3(104,253,119-180,693,127)x3		
		7pterq21.2(57,660-92,377,183)x2 hmz		
		10p15.3p12.3(111,955-20,876,388)x1		
		11q14.1q14.2(84,356,651-87,287,369)x1	*PICALM*	
		11q22.3-q23.1(107,154,315-112,417,074)x1		
		11q23.2(113,500,513-114,995,252)x1	*ZBTB16*	
		11q23.3(116,042,048-117,199,870)x1	*PAFAH1B2 PCSK7*	
		18q22.3q23(73,752,499-78,011,963)x1	*CTDP1*	
		21q22.11q22.3(32,065,821-48,042,513)x3		
26	Not done	(12)x3		
27	Not done	13q14.2q14.3(50,583,562-51,545,282)x0	*DLEU2 DLEU1 DLEU7*	I
				(bi-allelic)
28	Not done	6q14.3q24.3(87,140,674-148,617,199)x1		
		13q14.2q14.3(50,570,191-51,516,305)x1	*DLEU2 DLEU1 DLEU7*	I
		15q13.3q26.1(32,955,095-92,747,286)x1		
		17p13.3p11.2(103,469-18,820,457)x1		
		17q11.1q25.3(25,311,244-80,895,745)x3		

CNAs: Copy number alterations.

### High limit of detection and resolution of both microarray platforms as compared to FISH and MLPA

**Figure 1:**
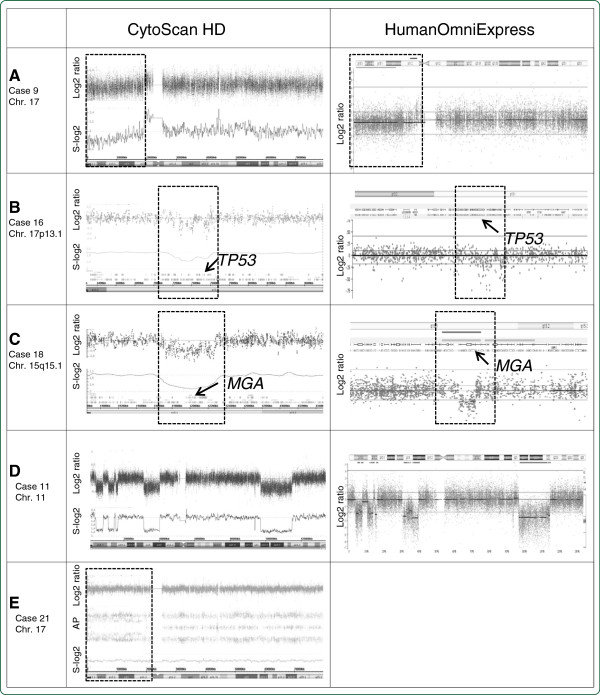
**Examples of microarray-based genomic profiles.** Microarray-based genomic profiles obtained using the CytoScan HD and HumanOmnioExpress platforms, showing log2 ratios, the log2 ratios smoothened over 10 probes (S-log2) and allele peaks (AP) (case 21 only). **A:** loss of the 17p region in case 9 (chromosome 17). **B:** Focal loss of the *TP53* gene in case 16 (showing the chromosome 17p13 region). **C:** Focal loss of the *MGA* gene in case 18 (showing chromosome 15q15.1). **D:** Chromothripsis of chromosome 11 in case 11. **E:** No abnormalities in the log2 ratio and smoothened log2 ratio in case 21. Instead of the 3 expected allele peaks (AA, AB and BB), a pattern showing mainly the AA and BB alleles is observed on the short arm of chromosome 17, indicating the CNLOH.

Microarray-based genomic profiling with the CytoScan HD Array and/or the HumanOmniExpress platform were applied to all 28 patients. For an unbiased comparison of the performance of both microarray platforms 9 patients (cases 7, 9, 10, 11, 16, 17, 18, 19 and 20) were evaluated on both microarray platforms in a fully blinded fashion, using identical interpretation criteria (see Methods section). By doing so, we found that both microarray platforms revealed exactly identical genomic aberrations (CNA and CNLOH). For representative examples see Figure 1.

For assessment of the limit of detection of the microarray platforms, 7 cases (cases 7, 9, 10, 11, 17, 20 and 24) were selected, since in these cases the genomic abnormalities were present in relatively low percentages of the cells (range 5-28%) as determined by FISH. All abnormalities present in more than 16% of the cells were readily detected by both microarray platforms, including 3 cases with a trisomy (cases 17 and 24 both with a trisomy 12 in 21% of the cells, and case 10 with a trisomy 8 in 28% of the cells), 3 cases (cases 7, 9 and 10) with loss of 17p in 16% to 21% of the cells (Figure 1A), and case 11 with loss of 13q14 in 28% of the cells. Case 20 was included in this study since 5% of its peripheral blood cells contained a bi-allelic loss of 13q14 as determined by FISH. Using the Cytoscan HD Array platform, this latter 1.7 Mb loss on 13q14 was barely detectable, whereas it was below detection level using the HumanOmniExpress Array platform.

For assessment of the resolution of both microarray platforms, case 16 was used since this case harbors focal loss of the *TP53* gene as determined by MLPA. This loss was not detected by the FISH probe used, since approximately half of the probe covered a non-deleted segment. Using both microarray platforms we found that the ~350 kb loss encompassing the *TP53* gene could readily be detected (Figure 1B).

### Abnormalities identified by microarray-based profiling but not by FISH or MLPA

Sixteen of the 28 patients (57%) carried additional genetic abnormalities (among which focal CNAs and CNLOH), not detected by FISH and/or MLPA. Twenty-four CNAs larger than 5 Mb, outside the loci tested by FISH and/or MLPA, were identified in 12 different cases (cases 3, 4, 7, 9, 10, 11, 12, 15, 21, 24, 25 and 28) (Tables 1 and 2). Although the clinical impact of these CNAs still has to be determined, recurrently affected regions such as gain of the short arm of chromosome 2 including the *MYCN* gene (cases 4, 9 and 25), loss of the long arm of chromosome 6 (cases 9, 12 and 28), and gains of chromosomes 18 and 19 (case 15) were noted. In addition, genomic complexity, defined as 3 or more >5 Mb CNAs, was observed in 11 cases (cases 3, 4, 7, 9, 10, 11, 12, 15, 21, 25, 28). Focal losses not observed by FISH and/or MLPA and below the level of cytogenetic resolution (<5 Mb), but containing cancer genes listed in (http://cancer.sanger.ac.uk/cancergenome/projects/census), were identified in 5 cases (cases 9, 16, 18, 21 and 25; Table 2). Case 16 harbors a focal *TP53* loss which was not identified by FISH (see above), but could be identified by MLPA and both microarray platforms (Figure 1B). Of interest, one patient (case 18) was found to harbor a recently identified recurrent loss on chromosome 15q15, which includes the *MGA* gene (Figure 1C), another patient (case 21) showed a recurrent loss on chromosome 10q24, which includes the *NFKB2* gene [5] and a third patient (case 25) showed a loss on chromosome 4q31 which encompasses the *FBXW7* gene [7]. In addition, focal CNAs involving the known cancer genes *SPECC1*, *WHSC1, TPR, FIP1L1, PICALM, ZBTB16, PAFAH1B2, PCSK7,* and *CTDP1* (cases 9, 18 and 25) were identified. Since their involvement in CLL has not been reported before, their clinical significance is still unclear.

CNLOH was detected in 5 cases (cases 3, 17, 20, 21 and 25) and was often found to coincide with several recurrently affected regions in CLL, including 13q14 in case 3 and 17p in case 21 (Figure 1E). Of interest, in this latter case a homozygous pathogenic *TP53* mutation was identified by targeted DNA sequencing (data not shown).

Previously, it has been suggested that the size of the 13q14 deletion may influence the clinical prognosis [8,9], i.e. patients with deletions encompassing the minimally deleted region (MDR) and smaller than <2 Mb in size (Type I deletions) were found to exhibit a better clinical prognosis as compared to patients with larger deletions often including the *RB1* locus (Type II deletions) [9]. A concomitant advantage of microarray-based genomic profiling is that the size of the 13q14 deletion can directly be delineated, whereby cases with Type I and Type II deletions can be discerned. In our panel, 7 cases (cases 2, 6, 8, 18, 20, 27 and 28) with a Type I and 6 other cases (cases 3, 4, 11, 16, 21 and 23) with a Type II deletion could be identified in this way (Table 2). In another case (11) we additionally observed alternating regions of gains, losses and a normal copy number status involving chromosomes 3 and 11 (Figure 1D), both fulfilling the definition of chromothripsis and reflecting genomic instability [10].

## Discussion

In the present study we evaluated the efficacy of microarray-based genomic profiling in comparison to routinely applied techniques such as FISH and MLPA in the genetic diagnosis of CLL. One important benefit of microarray-based genomic profiling is its ability to detect chromosomal aberrations not detected by routinely used targeted probe-based assays such as FISH and MLPA, but with clinical relevance. Although in the recent past CLL has been studied using a wide range of genomic microarrays [5,6,11-16], these studies were mainly focused on the putative prognostic significance of newly identified genomic alterations. The specific aim of our study was to assess the efficacy of two recently launched high resolution microarray platforms (i.e., CytoScan HD and HumanOmniExpress) in a clinical diagnostic setting, with special emphasis on its limit of detection and resolution.

From our results we conclude that both microarray platforms performed equally well with respect to detecting genomic alterations (both CNAs and CNLOH). This performance was assessed by applying identical interpretation criteria (see Methods section), thereby emphasizing the importance of applying uniform (international) interpretation criteria, especially when microarray-based genomic profiling will increasingly be used in clinical diagnostic settings.

Since in a routine clinical diagnostic setting parallel microarray-based assays using matched normal DNAs will not be feasible, we have established stringent interpretation criteria based on previous microarray studies performed on several types of hematological malignancies [17-19]. These interpretation criteria are set in such a way that non-tumor-related copy number variants and CNLOH are excluded. Only gross CNAs (>5 Mb) and CNLOH (>25 Mb) or those extending to the telomeres were considered as tumor-associated abnormalities. Focal CNAs were only considered when they encompassed (a) known tumor-related gene(s). In addition, we excluded variants present in a panel of healthy individuals identified by using the same microarray platforms (~250 for the HumanOmniExpress and ~1,000 for the CytoScan HD). This approach allows for the filtering of background noise such as calling errors and genomic polymorphisms and, at the same time, allows for the identification of recurrently affected genomic regions, as well as regions of potential clinical or biological relevance in CLL.

In order to evaluate the detection limit of the microarray platforms, 7 cases (7, 9, 10, 11, 17, 20 and 24) were selected in which the genomic abnormality was present in 5-28% of the cells as determined by interphase FISH. By doing so, both microarray platforms exhibited a high limit of detection, i.e., CNAs present in at least 16% of the cells could unambiguously be detected and, in addition, a bi-allelic loss of 13q14 present in only 5% of the cells could be observed on the CytoScan HD array platform. Although in the present study low-mosaic CNAs present in at least 16% of the cells are readily detected, others [6,12,15,16,20] have reported CNAs identified by FISH (ranging from 10% to ~40% of abnormal cells) that remained undetected using genomic microarrays. This may, at least in part, be explained by the microarray platforms used, which appeared to exhibit a lower limit of detection, the choice of the software packages used [14], or even false-positive FISH results [15].

Despite the fact that we performed microarray-based genomic profiling on whole peripheral blood samples, and not on (CD19) enriched cells, we have shown the feasibility of microarray-based profiling to detect genomic abnormalities in peripheral blood samples from CLL patients. This finding is not unexpected, as CLL is characterized by a clonal expansion of B cells in peripheral blood. Nonetheless, in laboratories that use microarray platforms with a lower limit of detection, an enrichment step for CD19-positive cells could improve the detection rate. An internal check for the presence of sufficient numbers of clonal B cells in the patient samples can be obtained by analysis of the microarray profiles for the *IGH, IGK* and *IGL* genes, for which copy number alterations reflect physiological events accompanied by somatic V(-D-)J assemblies of the *IGH*, *IGK* and *IGL* genes in the clonal B cell populations [21,22]. We emphasize the importance of detecting small CLL clones based on their putative clinical impact in CLL patients. As yet, there is still some controversy regarding the relevance of the size of a 17p deletion clone, in the range 10% to 25%, which could be associated with risk stratification and inferior outcome [23,24].

In 16 of the 28 CLL patients included, additional genomic alterations were identified by microarray-based profiling. Although the prognostic impact of many of these alterations awaits to be defined, an a priori clinical relevance may be assigned to at least some of them. It has previously been shown that the presence of a high number of CNAs (ranging from 1 to ≥3 abnormalities >5 Mb), referred to as genomic complexity, serves as an independent risk factor for disease progression [11,15,25]. In the present study genomic complexity (as defined by 3 or more aberrations ≥ 5 Mb) was detected in 11 cases, and was found to be associated with other high-risk features, such as 11q22 loss, 17p13 loss and *TP53* mutation in 9 of these 11 patients.

Eleven of the focal CNAs (smaller than 5 Mb and containing a tumor-related gene) were found to be recurrent, such as lesions involving the *RB1, LEU2, DLEU1, DLEU7, and TP53* genes, whereas other CNAs encompassed genes (i.e., *MGA, NFKB2* and *FBXW7*) known to play a role in CLL [5,7]. In one case (16) we identified a small focal *TP53* loss, which was not detected by FISH. This focal *TP53* loss was detected by both microarray platforms (Figure 1B) and by MLPA. These latter findings indicate that microarray-based profiling not only allows the genome-wide detection of genomic abnormalities, but also has a higher resolution for detecting clinical relevant focal lesions as compared to FISH and thus, is of added value. For the 8 non-recurrent CNAs detected involving tumor-related gene(s) (cases 9, 18 and 25), their role in the pathogenesis in CLL is as yet unknown.

Tumor-specific CNLOH was found in 5 cases, and in 2 of these cases the CNLOH involved regions affected by recurrent CNAs such as 13q14 and 17p13. In the patient with 17p CNLOH, this genomic aberration was associated with a homozygous *TP53* mutation. Our CNLOH observations are consistent with previously published data in which focal bi-allelic deletions in 13q14 and homozygous *TP53* mutations were found within larger CNLOH regions [5,25,26].

High resolution microarray-based genomic profiling allows the definition of the size of recurrently deleted regions in CLL. In this way we were able to discriminate between cases with a large 13q deletion involving the *RB1* gene (Type II deletion) (cases 3, 4, 11, 16, 21 and 23) associated with shorter time to treatment and overall survival, from those with smaller losses encompassing the *DLEU2, DLEU1* and *DLEU7* genes and some micro-RNAs (*MIR15A* and *MIR16-1)* (Type I deletion) (cases 2, 6, 8, 18, 20, 27 and 28) [8,9]. Microarray-based genomic profiling also allowed the detection of chromothripsis (case 11). This phenomenon was initially described in CLL as a new oncogenic event [10]. In an univariate analysis it has been shown that CLL patients with chromothripsis have an inferior outcome [5].

## Conclusion

We here show that both microarray platforms tested exhibit a high limit of detection and resolution to identify clinically relevant genomic aberrations, including those that escape routine FISH and/or MLPA-based analyses, in CLL. In our hands, CNAs present in only 16% of the cells as determined by FISH can unambiguously be identified. By applying similar interpretation criteria, results obtained from different microarray platforms are comparable. This opens up the possibility to fully replace the use of the current FISH panel by microarray-based profiling in all CLL patients. In addition, we show that microarray-based genomic profiling allows the detection of putative prognostic relevant abnormalities (i.e., focal *TP53* deletions, CNLOH of 17p, size of 13q14 deletions and genomic complexity), that would have remained undetected by routine FISH and/or MLPA procedures. The ultimate prognostic value of these novel genomic alterations requires further evaluation in prospective clinical trials.

## Methods

### Patient samples and DNA isolation

Blood or bone marrow samples were collected from 28 CLL patients from two different institutes (Radboud university medical center and University Medical Center Groningen). The diagnosis CLL was based on standard morphologic and immunophenotypic criteria [27]. To determine the limit of detection of both microarray platforms, 7 patients with low percentages of abnormal cells as determined by FISH were selected from this cohort. One aliquot of each blood or bone marrow sample was cultured for 24 hours and a standard cytogenetic cell preparation was prepared for FISH analysis. From a second aliquot DNA was extracted using a QIAamp DNA mini kit (Qiagen, Venlo, The Netherlands) or the Maxwell Instrument (Promega, Leiden, The Netherlands), both according to the instructions of the manufacturers.

### FISH analysis

The following commercially available probes were used for FISH: *ATM* (11q22), centromere 12, D13S319 (13q14) and *TP53* (17p13) (all from Abbott Molecular, Des Plaines, Illinois). FISH was performed according to the manufacturer’s specifications. At least 100 interphase nuclei were scored by two independent investigators. Overall, there was a perfect concordance in scoring between the two investigators. The cut-off values for both gains and losses were determined by statistical evaluation of FISH results from control tissues: for each probe the mean + 3 standard deviations of false positive nuclei was taken as the cut-off level.

### MLPA analysis

MLPA was carried out as described before [4] using two probe sets specifically designed for the detection of genetic aberrations in CLL, i.e., P037 and P038 (MRC-Holland, Amsterdam, The Netherlands). Amplified products were analyzed by capillary electrophoresis on an ABI 3730 genetic analyzer (Life Technologies, Carlsbad, USA). Data were normalized by dividing each probe’s peak area by the average peak area of the sample. This normalized peak pattern was divided by the average normalized peak pattern of all healthy control samples included in the same experiment. In a diploid situation, i.e., when two DNA copies are present in all cells, a relative copy number (RCN) of 1.0 is expected. When a deletion or duplication is present, the RCN will deviate towards 0.5 or 1.5, respectively.

### Genomic profiling and data analysis

Microarray-based genomic profiling was carried out in a blinded fashion using two different platforms; the CytoSan HD array platform (Affymetrix, Inc., Santa Clara, CA, USA) and the HumanOmniExpress12v1.0 array platform (Illumina Inc., San Diego, CA, USA). Hybridizations were performed according to the manufacturer’s protocols. The data obtained by the CytoScan HD array platform were analyzed using the Chromosome Analysis Suite software package (Affymetrix), and for the HumanOmniExpress12v1.0 platform data were analyzed using Nexus copy number software (Biodiscovery Inc., Hawthorne, CA, USA) using annotations of genome version GRCh37 (hg19).

### Interpretation of microarray data in CLL

For a comprehensive analysis of the microarray-based genomic profiling data we used a previously developed filtering pipeline, and its interpretation was performed using criteria adapted from [28]: (i) All segments larger than 5 Mb (resolution of conventional karyotyping), regardless of gene content, were denoted as true aberrations. (ii) All segments smaller than 5 Mb that coincided with known cancer genes (http://cancer.sanger.ac.uk/cancergenome/projects/census/ date of accession November 2012) were included. (iii) Since paired control DNA was not used, alterations that coincided with normal genomic variants were excluded. For this approach the publicly available database ‘Database of Genomic Variants’ (http://projects.tcag.ca/variation/ NetAfix version 32; date of accession February 2012) and, in addition, the in-house databases in which CNVs are stored from respectively ~1,000 healthy individuals run on the CytoScan HD platform and ~250 healthy individuals run on the HumanOmniExpress were used. (iv) Regions of copy-neutral loss of heterozygosity (CNLOH), also known as acquired uniparental disomy (UPD) were only considered if they were >25 Mb in size or if they extended towards the telomeres of the involved chromosomes, based on [18,19,29]. (v) Focal CNAs in the immunoglobulin genes were excluded from this study, since these lesions generally represent the B-cell clone with a rearranged immunoglobulin gene.

All the data were also visually inspected to define alterations present in a lower proportion of cells, and to eliminate alterations reported in regions with low probe density. Only aberrations fulfilling the above criteria were included in the genomic profiles, and were described according the standardized ISCN 2013 nomenclature system [30].

### Ethical consent

This study performed according to code for proper use of human tissue in the Netherlands as determined by the Federation of Medical Scientific Societies and in compliance with the Helsinki Declaration.

## Competing interests

All authors declare that they have no competing interests.

## Authors’ contributions

MJPLSK designed the experimental study and drafted the manuscript. EB, AGK and PJTAG participated in the design of the study and helped draft the manuscript. DOW, RP, TD and AS performed data analysis and helped draft the manuscript. MLW an MB carried out experiments and performed data analysis. All authors read and approved the final manuscript.
